# Antibacterial cellulose paper made with silver-coated gold nanoparticles

**DOI:** 10.1038/s41598-017-03357-w

**Published:** 2017-06-09

**Authors:** Tsung-Ting Tsai, Tse-Hao Huang, Chih-Jung Chang, Natalie Yi-Ju Ho, Yu-Ting Tseng, Chien-Fu Chen

**Affiliations:** 1 Department of Orthopaedic Surgery, Bone and Joint Research Center, Chang Gung Memorial Hospital and Chang Gung University College of Medicine, Taoyuan, 333 Taiwan; 2 Institute of Applied Mechanics, National Taiwan University, Taipei, 106 Taiwan; 3 Department of Chemistry, National Taiwan University, Taipei, 106 Taiwan

## Abstract

In this study, we investigated the antibacterial activity of silver-coated gold nanoparticles (Au-Ag NPs) immobilized on cellulose paper. Ag NPs are known to have strong antibacterial properties, while Au NPs are biocompatible and relatively simple to prepare. We made the Au-Ag NPs using a facile process called Ag enhancement, in which Au NPs serve as the nuclei for precipitation of a Ag coating, the thickness of which can be easily controlled by varying the ratio of the reactants. After synthesis, electron microscopy showed that the Au-Ag NPs displayed a core-shell structure, and that they could be successfully immobilized onto a cellulose membrane by heat treatment. We then investigated the antibacterial properties of this NP-coated cellulose paper against *E*. *coli* JM109. The inhibition rate, growth curve, and AATCC 100 activity test showed that cellulose paper coated with 15 nm Au-Ag NPs possessed excellent antibacterial activity against *E*. *coli* JM109. These results suggest that Au-Ag NPs immobilized on cellulose paper could be a valuable antibacterial technology for applications such as food packaging, clothing, wound dressings, and other personal care products.

## Introduction

Cellulose is the most abundant biopolymer on earth^1^ and has been used for a wide range of applications, such as filtration, food, medicine, pregnancy tests, cosmetics, and healthcare due to its low-cost, low-toxicity, hydrophilicity, biocompatibility, and flexibility^2–7^. Since cellulose is so commonly used in biomedical fields and food packaging, it is expedient that its antibacterial activity should be addressed^8, 9^.

There has been considerable interest in developing new techniques to impart antibacterial properties to cellulose paper for biochemical safety applications^10, 11^, particularly as there are many types of pathogenic bacteria that can have a negative impact on human health^12^. Among all the different kinds of antibacterial materials, researchers are increasingly turning to nanoscale organic and inorganic particles due to their unique combination of physical and chemical properties, which have been used in many biochemical applications, such as sensing, theranostics, drug delivery, and imaging^13–19^. In particular, researchers have examined the properties of inorganic metal nanoparticles (NPs), such as those made of silver (Ag), gold (Au), zinc, and copper^20–23^. Among these materials, Ag NPs have demonstrated excellent antibacterial activity through mechanisms involving the release of Ag^+^ ions that affect the replication of DNA^24^ or the collapse of the proton-motive force across the cytoplasmic membrane^25^. Direct growth of Ag NPs on the cellulose substrate to implement antibacterial function has also been realized^26^.

However, the overuse of Ag NPs also raises some health and ecological concerns. The dissolution of Ag NPs in water causes the material to slowly dissolve into ions on a time-scale of several days^27^, and cell toxicity increases with the quantity of silver ions in the dispersion. *In vitro* and *in vivo* studies have shown that Ag NPs are suspected of displaying toxicity effects in mammalian cells derived from the skin, liver, lung, brain, vascular system, and other organs^20, 28^. Even though there exist several commercially available products that feature Ag NPs to inhibit bacteria to treat cuts, scrapes, and burns, it is important to develop new materials that display lower toxicity and higher biocompatibility to produce antibacterial products that are safer for both people and the environment.

Conversely, Au NPs have been proven to possess high biocompatibility without acute cytotoxicity, and as a result, Au NPs have been extensively used for the development of new drug delivery and therapeutic methods^29, 30^. However, the biocompatibility of Au NPs also means they are not particularly antibacterial^31–33^.

In this work, we studied how Au and Ag NPs can be combined in cellulose paper to create a safer, yet still effective antibacterial material that is suitable for biomedical and food packaging purposes. Our aim was to coat Au NPs of varying size with Ag (Au-Ag NPs) using a procedure called “silver enhancement” that has been widely used in immune-chromatography to provide signal amplification and thus enhance the sensitivity of detection^34, 35^. The formed thin Ag shell can provide effective antibacterial properties by releasing Ag^+^ ions like Ag NPs, and the Au-Ag NPs also present a relatively eco-friendly and safer approach that is suitable for biomedical purposes. This antibacterial concept was adopted from a similar strategy by Richter *et al*.^36^ who used Ag^+^ ions infused in benign lignin-core NPs coated with cationic polyelectrolyte to provide a greener alternative to metallic Ag NPs.

By varying the amount of Au and Ag reactants used, we were able to control the Au NP size (15 nm and 20 nm), as well as the thickness of the Ag coating, specifically studying Au to Ag reactant volume ratios of 100/1 (Au-Ag_100/1_) and 1000/1 (Au-Ag_1000/1_)_._ In order to investigate the antibacterial effects of the Au and Au-Ag NPs using a more practical approach, we coated the synthesized NPs onto commercially available cellulose paper *via* a post-deposition method. Our findings showed that Au-Ag NPs made using a 100/1 ratio of Au to Ag reactants possessed excellent antibacterial activity against *E*. *coli* JM109.

## Results

### Preparation and Characterization of the Au and Au-Ag NPs and Their Immobilized on the Cellulose Paper

**Figure 1:**
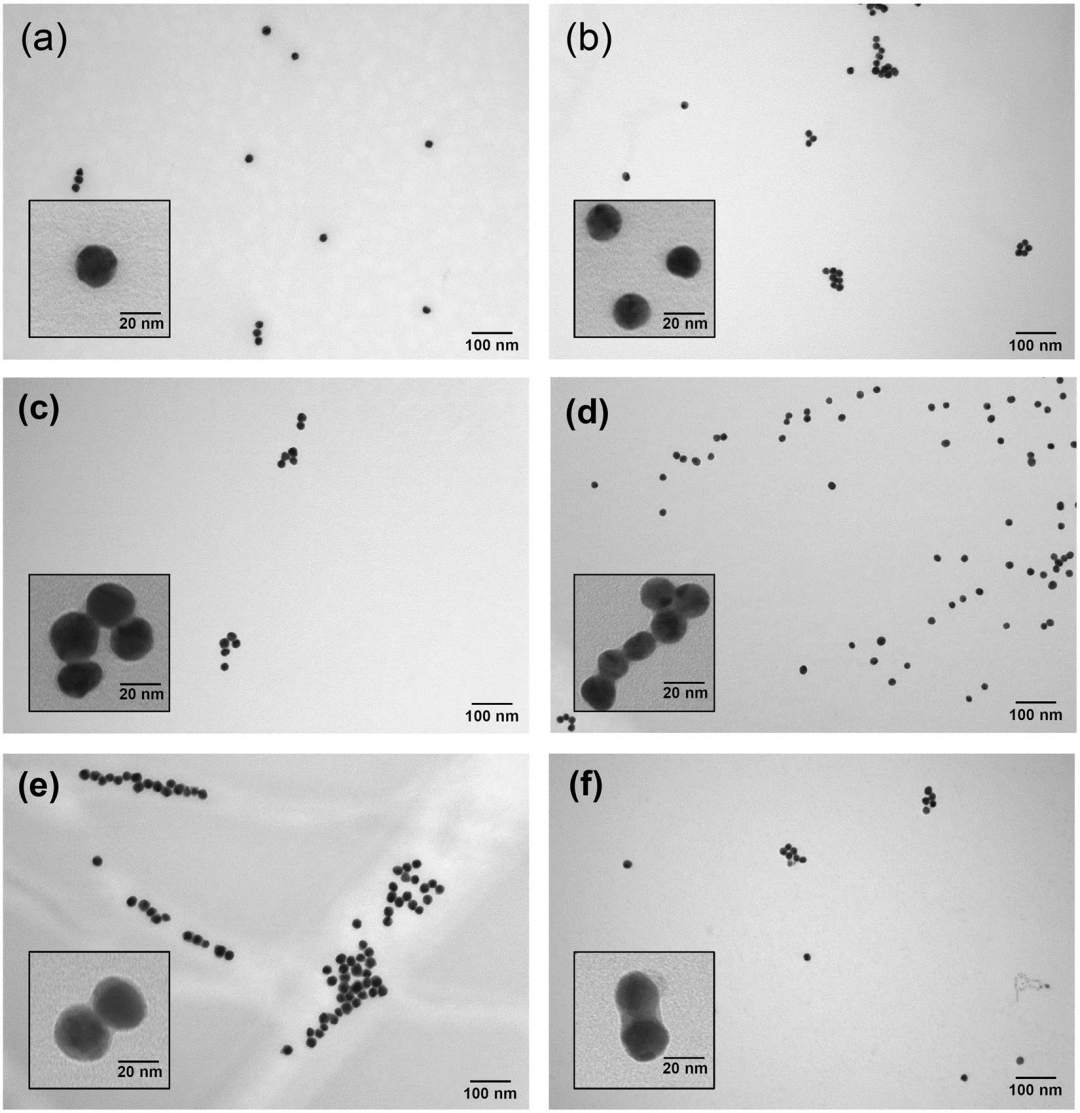
TEM images of the different Au and Au-Ag NPs. The 200,000× magnified images feature the (**a**) 20 nm Au NPs, (**b**) 15 nm Au NPs, (**c**) 20 nm Au-Ag_1000/1_ NPs, (**d**) 15 nm Au-Ag_1000/1_ NPs, (**e**) 20 nm Au-Ag_100/1_ NPs, and (**f**) 15 nm Au-Ag_100/1_ NPs. The inset of each figure is a 750,000× magnified image of the corresponding NPs.

We prepared Au-Ag NPs by depositing Ag on Au NP surfaces using different ratios of a diluted Ag enhancer solution, which enabled us to obtain different product compositions (see Methods for more details)^37–39^. In this way, we were able to study the effects of the NP diameter and Ag coating thickness on the activity of the resulting Au-Ag NP samples. The morphology and size of the synthesized NPs were measured using TEM (Fig. 1). Images of the 20 nm and 15 nm diameter Au NPs showed the materials featured good dispersion even after centrifugation and re-suspension (Fig. 1a,b). The size of the Au nuclei did not significantly change after we added the Ag enhancer, though we did observe the formation of bimetallic Au-Ag NPs using reactant ratios of 1000/1 and 100/1 (Fig. 1c–f), which displayed an obvious core-shell structure of a darker nucleus and a clearer shell (Fig. 1e,f), ~4 nm in thickness for the Au-Ag_100/1_ sample. The dark contrast of the nuclei corresponds to the Au NPs and the clear shells are due to the deposited Ag coating^40^. The material characterization results showed that different thicknesses of the Ag shells can be deposited on the Au NP surfaces without aggregation by varying the amount of silver nitrate. These observations were consistent with Bannerjee *et al*.’s^39^ work on Au@Ag core-shell NPs.

**Figure 2:**
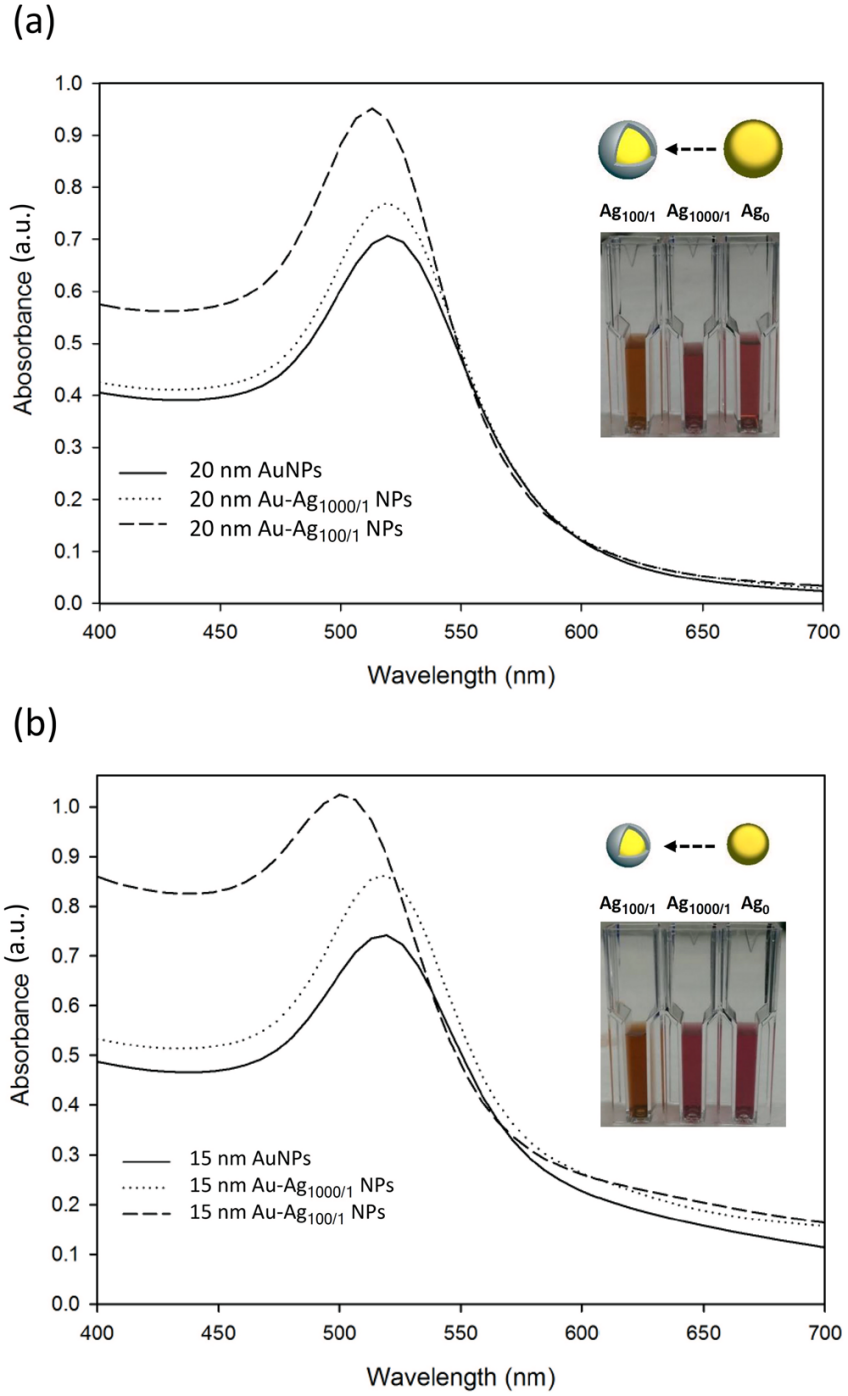
UV-Vis absorption spectra of different diameter Au and Au-Ag NPs. The plot shows the (**a**) 20 nm and (**b**) 15 nm Au and Au-Ag NPs at different ratios of Ag enhancement. The inset images show the solution color change after the Ag enhancement process.

To monitor the formation of Au-Ag NPs, we measured the UV-Vis absorption spectra of the materials prepared using different ratios of the Ag enhancement solution (Fig. 2). Before the addition of Ag, the characteristic peaks of the Au NPs were centered at 520 nm and 516 nm for the 20 nm and 15 nm diameter samples, respectively (Fig. 2a,b). The appearance of a single absorption band corresponding to each Au NP sample indicates the particles were homogeneous^40^. These results also match the characteristic surface plasmon resonance of Au NPs^41^. After the addition of the Ag enhancement solution, the peaks of the 15 nm and 20 nm Au-Ag_1000/1_ NP samples slightly blue-shifted 2–3 nm, while the absorption peaks of the 15 nm and 20 nm Au-Ag_100/1_ NP samples each shifted to 500 nm. We have also conducted dynamic light scattering (DLS) analyses to confirm the Ag enhancement process did not lead to the fusion of the NPs (Table S1). From these DLS results, we found that the two sets of NPs possessed a mean diameter of around 15 nm and 20 nm, thus confirming that the Ag enhancement process did not cause the NPs to fuse.

**Figure 3:**
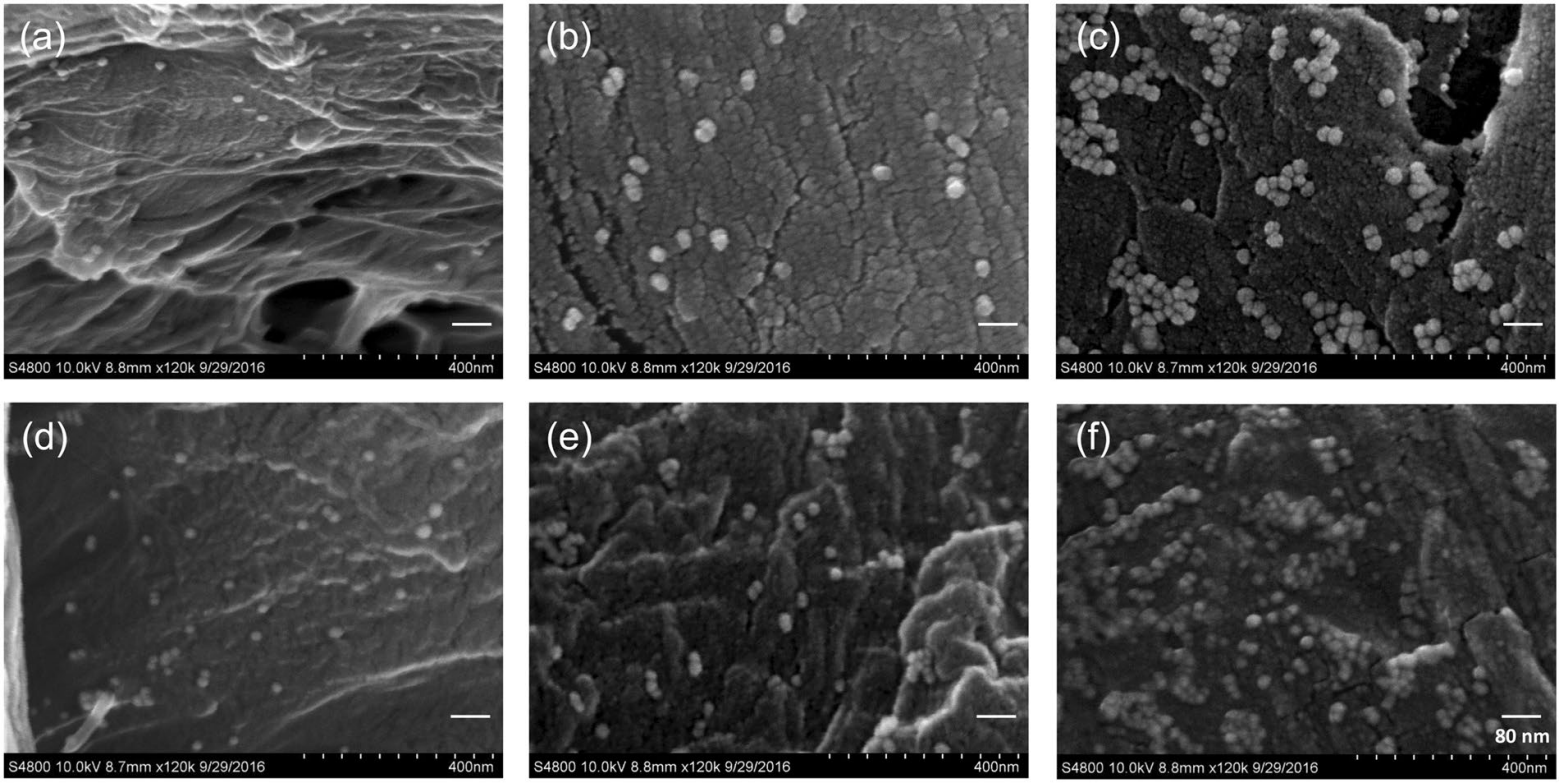
SEM images of the different Au and Au-Ag NPs on cellulose paper. The bright spots in the images are NPs, specifically (**a**) 20 nm Au NPs, (**b**) 20 nm Au-Ag_1000/1_ NPs, (**c**) 20 nm Au-Ag_100/1_ NPs, (**d**) 15 nm Au NPs, (**e**) 15 nm Au-Ag_1000/1_ NPs, and (**f**) 15 nm Au-Ag_100/1_ NPs.

Next, we examined the morphology, structure, and size of the Au and Au-Ag NPs after they had been pipetted onto the cellulose paper. Using SEM imaging, we determined that the Au NPs on cellulose paper were monodisperse (Fig. 3a,d). However, after the Ag treatment, the resulting Au-Ag NPs tended to aggregate on the cellulose fibers, particularly for samples made using the higher 100/1 ratio of the Ag reactant (Fig. 3b,c,e,f). These SEM images further confirm that Ag deposition causes the previously monodisperse Au NPs to aggregate and form Au-Ag NP clusters, as has been previously observed in other work^42^. These results are also corroborated by the transmission electron microscopy (TEM) measurements, which showed the core-shell structure particles closely grouped together (inset images of Fig. 1c,d,e,f).

One way to study the application of cellulose as an antibacterial substrate is to directly synthesize the NPs onto the paper itself ^26^. However, direct synthesis in this manner can cause variability in the NP morphology, which can make it difficult to obtain repeatable results. In this study, the NPs were immobilized on cellulose paper by pipetting the colloid solutions onto the substrates and heating the samples at 37 °C for 1 h to completely dry out the composite and increase adhesion between the particles and the fibers. We checked the color of the paper substrate after antibacterial testing, which involved 24 h of contact with a dilute bacterial suspension, and found that the color of the paper remained red, corresponding to the color of the immobilized NPs (Fig. S1).

**Figure 4:**
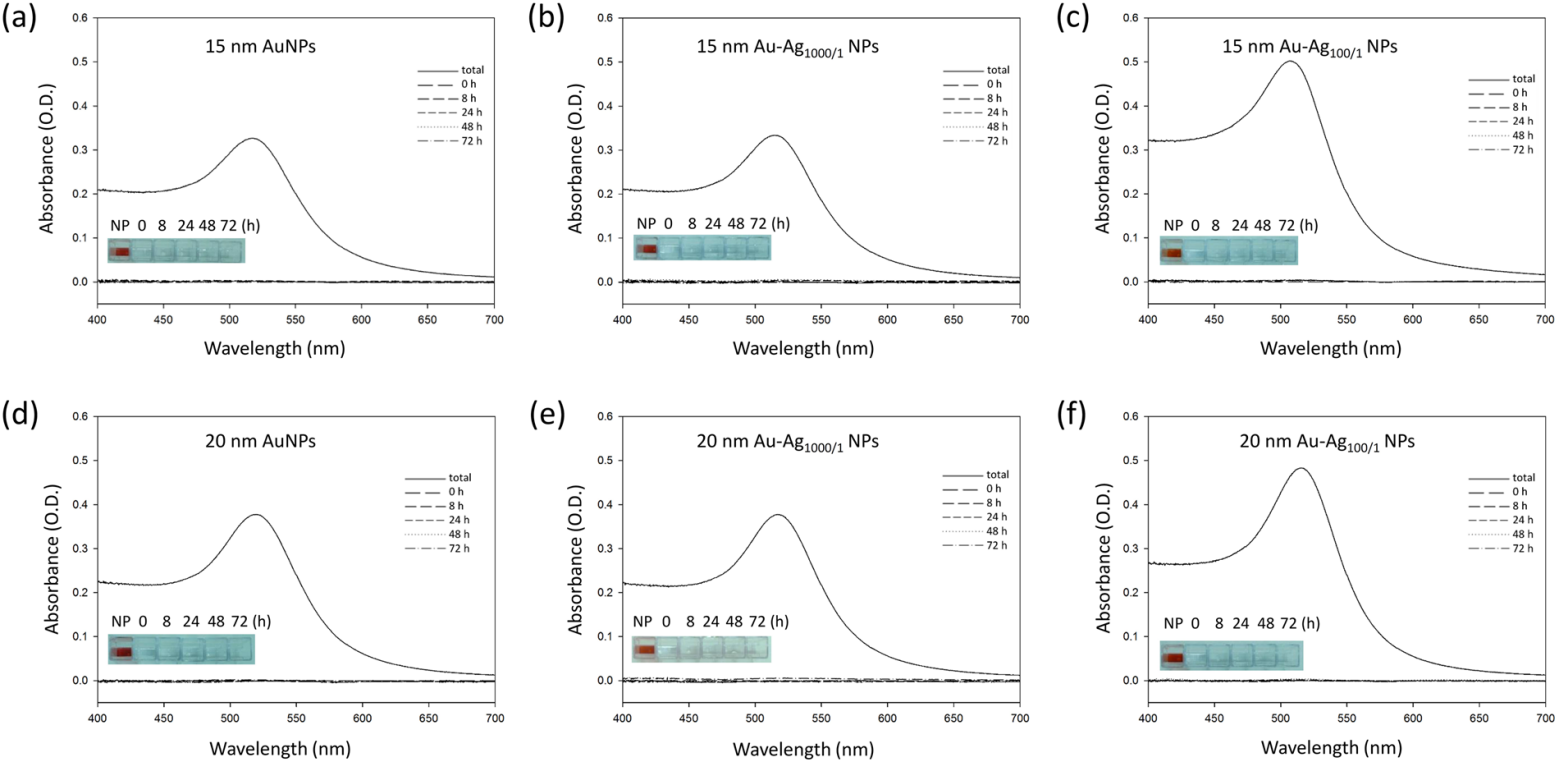
UV-Vis absorption spectra of the NP detachment tests. The solutions were collected at 0 h, 8 h, 24 h, 48 h, and 72 h. The absorption spectra were measured to determine the amount of NPs in solution. The inset images are of the original NP stock solutions used to make the NP-paper composites (“NP”) and the water solutions collected after the paper samples had been immersed for 0 h, 8 h, 24 h, 48 h, and 72 h. The results show that no significant NP signal can be seen in solution during this time period.

In order to verify whether the Au-Ag NPs were sufficiently attached to the substrate, we immersed the NP-cellulose paper in water and then performed TEM and UV-Vis absorption spectroscopy on those solutions after the NP-cellulose paper had been immersed in water for 0 h, 8 h, 24 h, 48 h, and 72 h. Absorption measurements of the original NP stock solutions that were used to make the paper samples were also taken (“NP”). The UV-Vis absorption spectra showed that no significant signal could be observed from the NP-cellulose paper after immersion in water for 72 h (Fig. 4). The solutions also remained visibly clear during this time period. In order to confirm the results, we used TEM to observe the contents of this water after NP-cellulose paper immersion for 72 h (Fig. S2). Despite the lack of change in the absorption spectra, the TEM images showed a low density of dispersed NPs. We estimated the concentration of the released NPs by comparing these results with TEM images and absorbance spectra of the calibration curves of the prepared NP solutions. Based on the visual similarity of NP densities, it appears that approximately 4 pM of the immobilized Au-Ag_100/1_ NPs was released from the cellulose after 72 h. Therefore, even with the heat treatment method to immobilize the NPs on the cellulose paper surface, the material still loses ~1% of the attached NPs after 72 h of immersion in water. However, this result also shows that the vast majority of the NPs remain immobilized on the cellulose paper when contacted with a solution-phase medium.

### Antibacterial Tests

**Figure 5:**
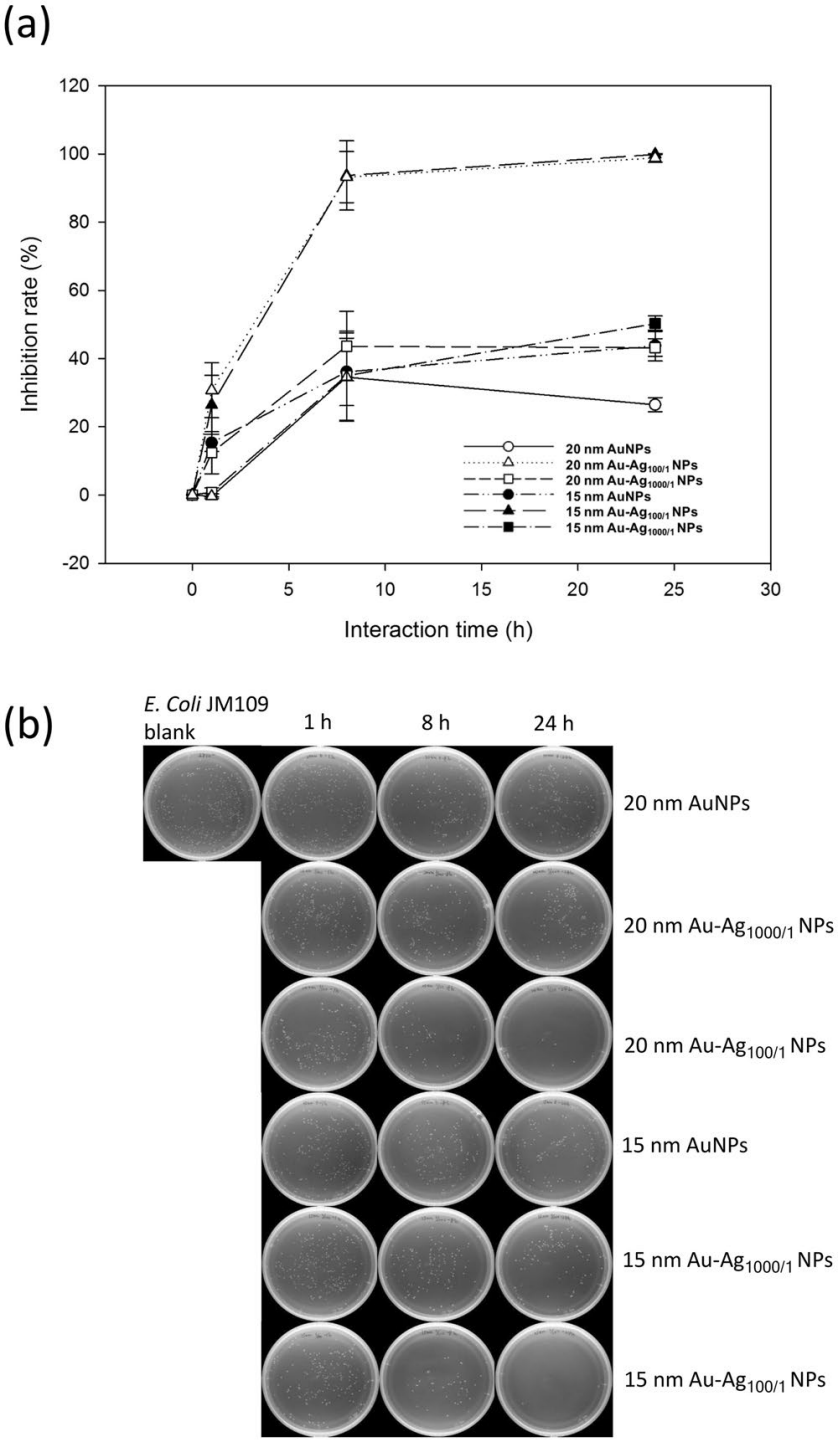
The antibacterial effects of different Au and Au-Ag NPs on *E*. *coli*. The (**a**) inhibition ratios of the bacterial growth and (**b**) typical photographs of the bacterial colonies formed after the Au, Au-Ag_1000/1_, and Au-Ag_100/1_ NP-cellulose paper samples had reacted with *E*. *coli* for 1, 8, and 24 h (n = 3).

**Figure 6:**
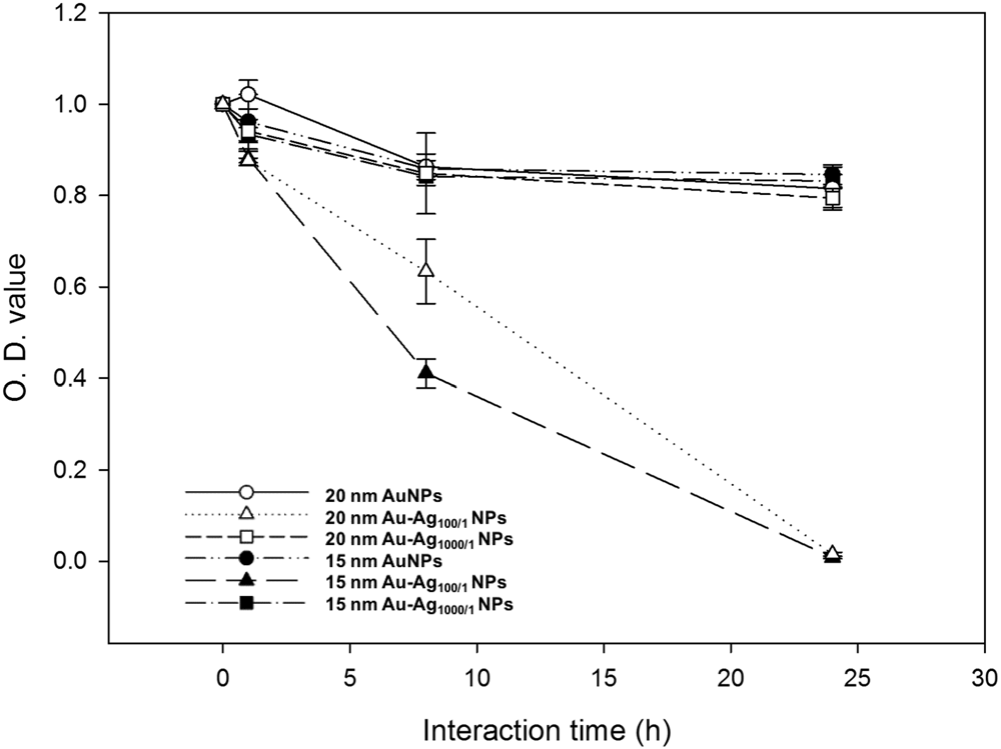
The survival curve of *E*. *coli* exposed to Au and Au-Ag NPs. *E*. *coli* were continuously exposed to different NP-coated cellulose paper for 24 h. The data is plotted as the percentage of surviving cells compared to the untreated controls (n = 3).

**Figure 7:**
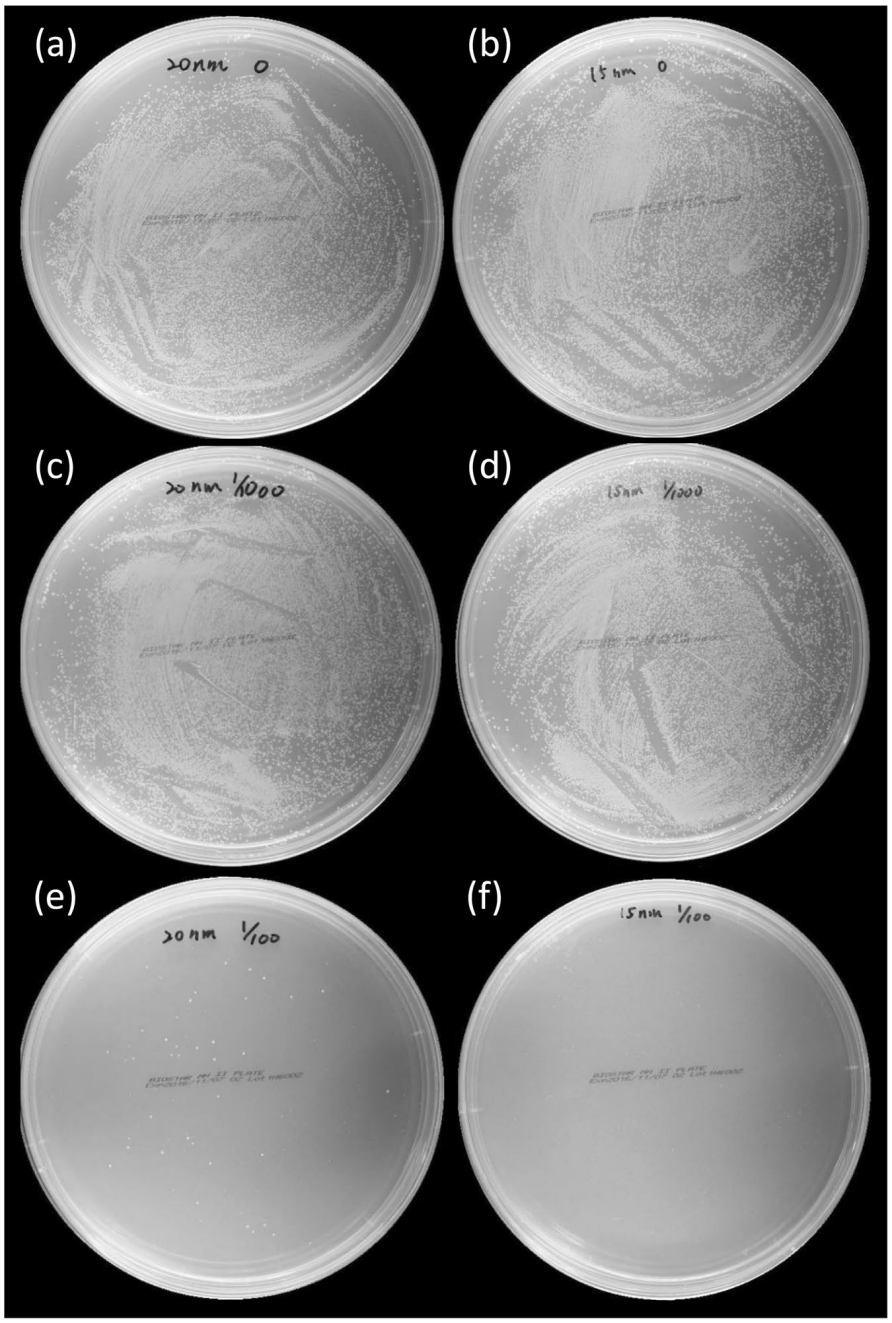
The antibacterial activity against *E*. *coli* on cellulose paper. The AATCC 100 antimicrobial fabric test was used to assess the antibacterial activity of the following NPs on cellulose paper, including (**a**) 20 nm Au NPs, (**b**) 15 nm Au NPs, (**c**) 20 nm Au-Ag_1000/1_ NPs, (**d**) 15 nm Au-Ag_1000/1_ NPs, (**e**) 20 nm Au-Ag_100/1_ NPs, and (**f**) 15 nm Au-Ag_100/1_ NPs.

After immobilizing Au and Au-Ag NPs on the cellulose paper, we investigated the composite materials’ antibacterial activity by combining these paper samples with *E*. *coli* JM109 in solution and measuring the inhibition and survival rates of the bacteria. We also determined the antibacterial activity of the Au-Ag NP paper composite by adapting the AATCC 100 activity test^43^, which is normally used to determine the antimicrobial properties of fabrics. The results of the antibacterial activity of the Au and Au-Ag NP samples deposited on cellulose paper are shown in Figs 5–7. Figure 5 shows the inhibition rate of *E*. *coli* JM109. After 1 h of *E*. *coli* incubation with the 15 nm and 20 nm Au NPs, we observed no significant reduction of the *E*. *coli* JM109 population. Our Au NPs feature citrate ligands, and thus this result is consistent with the previous report that anionic Au NPs are less toxic compared to those that are cationic^44^. We also observed a similar result for the Au-Ag_1000/1_ NP sample. For the 15 nm and 20 nm Au-Ag_1000/1_ NP samples, the reduction rate of *E*. *coli* JM109 population was just 0.16% and 16.75%, respectively. On the other hand, there was a 28% and 36% inhibition rate of *E*. *coli* JM109 for the 15 nm and 20 nm Au-Ag_100/1_ NPs, respectively. After 8 h, the inhibition rate for the 15 nm and 20 nm Au-Ag_1000/1_ NP samples plateaued at approximately 40%. In contrast, the inhibition rates of the 15 nm and 20 nm Au-Ag_100/1_ NP samples reached 100% after 24 h. These results demonstrate that when we increased the ratio of the Ag enhancer from 1000/1 to 100/1, the antibacterial activity of the NP-coated cellulose paper was more effective. This would suggest that the Ag shell is responsible for the antibacterial action, most likely due to the release of Ag^+^ ions from the surface of the Au-Ag NPs^45–47^.

Figure 6 shows the survival curves of the *E*. *coli* JM109 populations after exposure to the NP-coated cellulose samples, which we measured using the O.D. of the *E*. *coli* JM109 solution versus the sample interaction time. During the first hour, the 15 nm and 20 nm Au NPs coated on cellulose paper did not show significant antibacterial activity. When the interaction time was increased to 8 h, a similar bacterial density (approximately 0.8 to 0.9 O.D.) with no obvious antibacterial activity was observed for both the Au and Au-Ag_1000/1_ NPs. However, for the 15 nm and 20 nm Au-Ag_100/1_ NPs, only ~20% of the bacteria survived at this point. These results can be attributed to the antibacterial effects of the composition of the Au-Ag NPs. Ag NPs naturally bind to the bacterial membrane and disrupt the integrity, and Ag ions attach to essential biological molecules to inhibit the growth of bacteria^23, 48^. Moreover, the 15 nm Au-Ag_100/1_ NPs displayed stronger antibacterial activity compared to the 20 nm Au-Ag_100/1_ NP sample due to the fact that smaller NPs can more easily reach the nuclear content of the bacterial cells and the greater relative surface area of the Ag coating increases exposure to the bacteria^49, 50^. At 24 h, the Au-Ag_100/1_ NPs on cellulose paper resulted in 0% survival rate of the bacteria, while both the Au and Au-Ag_1000/1_ NP paper samples showed a limited effect of ~80% survival compared to the initial O.D. of the bacteria in solution.

We also used a modified version of the AATCC 100 antimicrobial fabric test to investigate the antibacterial activity of cellulose paper that had been coated with Au and Au-Ag NPs. To perform this test, we added 20 µL of an *E*. *coli* JM109 suspension onto NP-coated cellulose-based paper discs. After 24 h incubation, we then soaked the paper discs in isotonic sodium chloride and then spread 10 µL of this soaking solution onto the agar plates. The viable bacterial counts recovered from the cellulose paper before and after incubation are shown in Fig. 7. After 24 h of incubation, there was only a limited reduction in the viable *E*. *coli* JM109 population that had been loaded on cellulose paper pre-coated with Au (Fig. 7a,b) and Au-Ag_1000/1_ NPs (Fig. 7c,d). However, there was a nearly 100% reduction when the 15 nm Au-Ag_100/1_ NP sample was used (Fig. 7f). The 20 nm Au-Ag_100/1_ NPs also displayed a strong, though weaker reduction in viable *E*. *coli* bacteria compared to the 15 nm Au-Ag_100/1_ NPs (Fig. 7e). Therefore the Au-Ag_100/1_ coated cellulose paper demonstrated excellent antibacterial activity against *E*. *coli* JM109. These results were also consistent with the size-dependent antibacterial activity we had previously observed in the bacterial growth curve and suggest that the combination of these materials possesses the potential for more environmentally-friendly commercial antibacterial applications, such as food packaging, clothing, wound dressings, and other personal care products.

## Discussion

NPs have been used against many diseases that are caused by bacteria and viruses^51^. Herein, we present different sizes and compositions of Au and Au-Ag NPs deposited on cellulose paper to examine the antibacterial activity of the resulting composites. Based on the results, we demonstrated that Au-Ag_100/1_ and Au-Ag_1000/1_ NP-coated cellulose paper can reduce the growth of *E*. *coli*, with 15 nm Au-Ag_100/1_ NPs showing the strongest inhibitory effects. To date, there are only a few studies done on the activity of NP-coated cellulose paper toward bacterial pathogens^26, 52, 53^. Our results indicate that cellulose paper deposited with Au-Ag NPs have the potential to serve as effective antimicrobial products in the near future. Further studies on other Gram negative bacteria, such as *Staphylococcus aureus* and *Pseudomonas aeruginosa*, will be needed in order to verify whether those organisms will also develop resistance toward these Au-Ag NPs. It will also be necessary to examine the cytotoxicity and biocompatibility of these nanomaterials toward human cells before proposing their therapeutic use in cellulose composites.

## Methods

### Chemicals

Methanol (99%), 2-propanol (99%), ethanol (99%), trisodium citrate (>99%), hydrogen tetrachloroaurate (III) trihydrate (99%), and the Silver Enhancer Kit were purchased from Sigma-Aldrich (St. Louis, MO). *E*. *coli* JM109, LB agar, and LB broth were purchased from Geneaid Biotech Ltd. (New Taipei City, Taiwan), Athena Environmental Sciences, Inc. (Baltimore, MD), and AMRESCO LLC (Solon, OH), respectively. We used ultrapure water (18.2 mΩ·cm) throughout the experiments, which was filtered through a Milli-Q system (Millipore, Milford, MA). An isotonic sodium chloride solution (0.9%) was obtained from Taiwan Biotech Co., LTD. (Taoyuan, Taiwan). Whatman grade 1 chr cellulose chromatography paper was purchased from GE Healthcare (Little Chalfont, UK). Cellulose based TaxoTM blank paper discs were bought from BD, Becton Dickinson and Company (Franklin Lakes, NJ). Mueller Hinton II agar plates were ordered from Biostar Microtech Int’l Corp. (New Taipei City, Taiwan).

### Instrumentation and Characterization

TEM (H7500, Hitachi High-Technologies, Tokyo, Japan) was used to verify the size and morphology of the synthesized Au and Au-Ag NPs, and also to confirm the immobilization of the NPs on the cellulose paper. The distribution of the Au and Au-Ag NPs on cellulose chromatography paper was measured using SEM (JSM-6700F, JEOL, Tokyo, Japan). Au, Au-Ag NPs, and their immobilization on cellulose paper was also characterized with a UV-Vis spectrometer (Cintra 10e, GBC, Victoria, Australia). A zetasizer (Nano-HT, Malvern, UK) was employed to record the DLS measurement of the NPs in solution. An Orbital Shaking Incubator 740 (Cherng Huei Co., LTD., New Taipei City, Taiwan) was used for the bacterial cultures. An Ultrospec 10 Cell Density Meter (Amersham PLC, Little Chalfont, UK) was used to determine the optical density (O.D.) of the bacteria suspensions.

### Preparation of the Au and Au-Ag NPs

We synthesized Au NPs using the Turkevich method^54^. 20 nm diameter Au NPs were prepared as follows: 30 mL of 0.01 wt% tetrachloroauric acid in a 100 mL beaker was heated to boiling with vigorous stirring. Then 1 mL of 1 wt% trisodium citrate dihydrate was added quickly, which resulted in the color of the solution changing from yellow to black and then to red. We continued to heat and stir the solution for another 10 min and then cooled the sample in a water-ice bath for 30 min. The 15 nm Au NPs were prepared using the same method except we added 2 mL of trisodium citrate dihydrate.

To obtain different thicknesses of the Ag coatings on the Au-Ag NPs, we first prepared a fresh 1:1 mixture of the silver salt (solution A) and initiator (solution B) from the Silver Enhancer Kit (SE100, Sigma-Aldrich, St. Louis, MO). We then added the silver enhancer mixture to the Au NP solutions (3 O.D.) at a ratio of 100/1 (Au-Ag_100/1_: 10 µL silver enhancer mixture to 1000 µL of the Au NP solution) and 1000/1 (Au-Ag_1000/1_: 1 µL silver enhancer mixture to the 1000 µL Au NP solution). In this method, Au NPs in the presence of Ag (I) ions and a reducing agent act as catalysts to reduce the Ag (I) ions into metallic Ag. The metallic Ag is then deposited onto the Au to enlarge the particles^39, 55^. The mixtures were then incubated at room temperature for 30 min. After centrifugation at 6000 g for 25 min, we discarded the supernatant and re-suspended the pellets in ultrapure water. After synthesis, we characterized the size and morphology of the resulting Au-Ag NPs, as well as Au NP controls, using transmission electron microscopy (TEM), scanning electron microscopy (SEM), and ultraviolet-visible (UV-Vis) absorption spectroscopy. The samples were stored at 4 °C before the antibacterial tests.

In order to verify whether the Au-Ag NPs were immobilized on the cellulose paper, we first pre-coated 70 μL of the six different NP solutions, including 15 nm and 20 nm diameter Au NPs, Au-Ag_1000/1_, and Au-Ag_100/1_, on pieces of 1 cm^2^ cellulose paper and allowed them to heat at 37 °C for 1 h. For each of the six different synthesized NPs, we prepared twelve of the pre-coated cellulose paper samples and then placed them in 4 mL of ultrapure water. At 0 h, 8 h, 24 h, 48 h, and 72 h, 800 µL of the solution was collected and monitored with UV-Vis absorption spectroscopy and TEM imaging to determine the amount of Au-Ag NPs that had detached from the paper substrates.

### Antibacterial Testing (Inhibition Rate/ Survival Curve)

We selected a single colony of *E*. *coli* JM109 from LB agar plates and inoculated it in 5 mL LB broth. The bacterial culture was then grown overnight and later centrifuged at 2500 g for 10 min, followed by dilution to 1.0 × 10^4^ bacteria/mL in isotonic sodium chloride solution.

We pre-coated the 6 mm BD Taxo blank paper discs with 20 µL of the 6 different NP solutions (15 nm and 20 nm diameter Au NPs, Au-Ag_100/1_, and Au-Ag_1000/1_), which was just enough solution to wet the disc. We then dried the samples at 37 °C for 1 h before soaking each one in 2 mL of diluted bacterial suspension by shaking at 37 °C and 150 rpm in an incubator. At 0, 1, 8 and 24 h, we spread 20 µL of diluted bacterial solution from each sample onto 10 cm LB agar plates, which we incubated at 37 °C overnight. Afterwards, we measured the number of bacterial colonies on the plate. The plates were photographed and the quantitative analysis of the antibacterial effects of the different NPs was performed using the Promega Colony Counter app (Madison, WI, US). We counted the number of the colonies and then calculated the inhibition rate (IR) of the bacterial growth using the following equation:

1 $${\rm{IR}}( \% )=({{\rm{N}}}_{0}-{\rm{N}})/{{\rm{N}}}_{0}\times 100 \% $$

in which N_0_ and N were the number of the colonies detected on the blank plate made using DI water and the plates that had been made from solutions of the 6 different samples, respectively.

At the same 1, 8, and 24 h intervals, we also removed an additional 20 µL of diluted bacterial solution from each NP sample which was added to 2 mL LB broth and incubated at 37 °C overnight to determine the bacterial growth by measuring the bacterial density at OD_600_ using the Ultrospec 10 Cell Density Meter.

### Antibacterial Testing (AATCC 100)

We used an antibacterial testing method modified from the AATCC 100 antimicrobial testing protocol. In brief, a single colony of *E*. *coli* JM109 from the LB agar plates was selected and inoculated in 5 mL LB broth. The culture was then grown overnight. Next, we diluted the bacterial culture to 1.0 × 10^8^ bacteria/mL in LB broth. Then 20 µL of the diluted bacterial suspension was loaded onto BD Taxo blank paper discs that had been pre-coated with 20 µL of 6 different NP solutions and dried at 37 °C for 1 h. After 24 h incubation at 37 °C, we soaked each NP-coated paper disc sample in 200 µL isotonic sodium chloride solution, which was then vortexed. Next, we spread 10 µL of the soaking solutions from each sample onto 15 cm Mueller Hinton II agar plates and photographed the plates after incubation at 37 °C overnight.

## Electronic supplementary material

Electronic Supplementary Information
