# Supplementary material for: Antibacterial cellulose paper made with silver-coated gold nanoparticles
Source: Sci Rep. 2017 Jun 9;7:3155. doi: 10.1038/s41598-017-03357-w (PMC5466604; doi:10.1038/s41598-017-03357-w)
Supplement: Supplementary file 1 — Electronic Supplementary Information [file 41598_2017_3357_MOESM1_ESM.pdf]

Electronic Supplementary Information for

Antibacterial Cellulose Paper Made with Silver-Coated Gold Nanoparticles

Tsung-Ting Tsai[1], *Tse-Hao Huang*[12], *Chih-Jung Chang*[1], *Natalie Yi-Ju Ho*[1], *Yu-Ting Tseng*[3] & *Chien-Fu Chen*[2*]

[1]Department of Orthopaedic Surgery, Bone and Joint Research Center, Chang Gung Memorial Hospital and Chang Gung University College of Medicine, Taoyuan 333, Taiwan.

[2]Institute of Applied Mechanics, National Taiwan University, Taipei 106, Taiwan.

[3]Department of Chemistry, National Taiwan University, Taipei 106, Taiwan.

Table S1. DLS measurements of the Au, Au-$Ag_{100/1}$, and Au-$Ag_{1000/1}$ NPs.

	AuNPs	Au-$Ag_{100/1}$ NPs	Au-$Ag_{1000/1}$ NPs
15 nm	18.17 ± 4.2 nm	17.26 ± 5.6 nm	17.72 ± 4.9 nm
20 nm	22.83 ± 5.0 nm	22.69 ± 6.8 nm	22.03 ± 4.3 nm

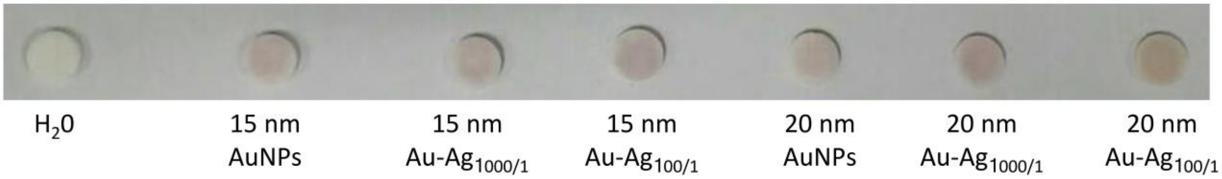

Figure S1. The color of the paper substrates immobilized with various NPs after 24 h of contact with a diluted bacterial suspension. After this test, the color of the paper substrates remained red, suggesting the NPs largely remained attached to the cellulose fibers.

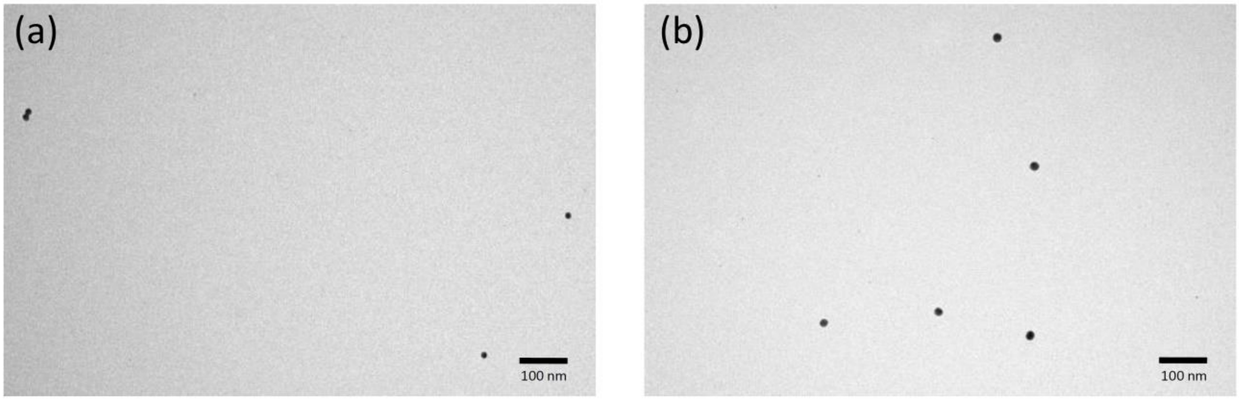

Figure S2. TEM images of the water solution after the (a) 15 nm and (b) 20 nm Au-$Ag_{100/1}$ NP-paper composites had been immersed for 72 h. A low density of detached NPs can be seen.
